# Comparative Genomics of *Botrytis cinerea* Strains with Differential Multi-Drug Resistance

**DOI:** 10.3389/fpls.2016.00554

**Published:** 2016-04-28

**Authors:** Michael Chatzidimopoulos, Fotis Psomopoulos, Emmanouil E. Malandrakis, Ioannis Ganopoulos, Panagiotis Madesis, Evangelos K. Vellios, Pavlina Drogoudi

**Affiliations:** ^1^Laboratory of Plant Pathology, Department of Agriculture, Crop Production and Rural Environment, University of ThessalyVolos, Greece; ^2^Department of Electrical and Computer Engineering, Aristotle University of ThessalonikiThessaloniki, Greece; ^3^Department of Ichthyology and Aquatic Environment, University of ThessalyVolos, Greece; ^4^Centre for Research and Technology Hellas, Institute of Applied BiosciencesThessaloniki, Greece; ^5^Department of Deciduous Fruit Trees in Naoussa, Institute of Plant Breeding and Plant Genetic Resources, Hellenic Agricultural Organization ‘Demeter’Naoussa, Greece

**Keywords:** *Botrytis cinerea*, anilinopyrimidines, whole genome sequencing, multi-drug resistance, next-generation sequencing (NGS)

## Introduction

*Botrytis cinerea* is a ubiquitous fungus difficult to control because it possess a variety of attack modes, diverse hosts as inoculum sources, and it can survive as mycelia and/or conidia or for extended periods as sclerotia in crop debris. For these reasons the use of any single control measure is unlikely to succeed and a combination of cultural practices with the application of site-specific synthetic compounds provide the best protection for the crops (Williamson et al., 2007). However, the chemical control has been adversely affected by the development of fungicide resistance. The selection of resistant individuals in a fungal population subjected to selective pressure due to fungicides is an evolutionary mechanism that promotes advantageous genotypes (Walker et al., 2013). High levels of resistance to site-specific fungicides are commonly associated with point mutations. For example the mutations G143A, H272R, and F412S leading to changes in the target proteins CytB, SdhB, and Erg27 are conferring resistance of the pathogen to the chemical classes of QoIs, SDHIs, and hydroxyanilides, respectively (Leroux, 2007). Multidrug resistance is another mechanism associated with resistance in *B. cinerea* which involves mutations leading to overexpression of individual transporters such as ABC and MFS (Kretschmer et al., 2009). This mechanism is associated with low levels of resistance to multiple fungicides including the anilinopyrimidines and phenylpyrroles. However, a subdivision of gray mold populations was found to be more tolerant to these two classes of fungicides (Leroch et al., 2013).

Previous reports have clearly demonstrated that the resistance to anilinopyrimidines has a qualitative, disruptive pattern, and is monogenically controlled (Chapeland et al., 1999). In order to elucidate the mechanism of the resistance, the whole genome of three different samples (gene pools) was sequenced, each containing DNA of 10 selected strains of the same genotype regarding resistance to seven different classes of fungicides including anilinopyrimidines. This report presents the publicly available genomic data.

## Materials and methods

### Isolation–determination of fungicide resistance profile

Pure cultures of *B. cinerea* were obtained on sterilized PDA media from infected lettuce plants by slight touching a flamed wire loop onto a freshly sporulating lesion. All isolates obtained from lettuce plants in a commercial lettuce glasshouse located at Krokion, Magnesia, Greece on February 26th, 2012. From each sample a single isolate was made. For all purposes single-spore isolates properly prepaired. The sensitivity of the isolates to the fungicides fenhexamid (class: Hydroxyanilides-Hyd), pyraclostrobin (class: Quinone outside Inhibitors-QoIs), boscalid (class: Succinate De-Hydrogenase Inhibitors-SDHIs; Bos), cyprodinil (class: Anilinopyrimidines-Ani), fludioxonil (class: Phenylpyrroles-Phen), carbendazim (class: Benzimidazoles-Ben), and iprodione (class: Dicarboximides-Dic) was determined by the point inoculation method using the discriminatory concentrations of each fungicide as defined by Chatzidimopoulos et al. (2013). The isolates were then classified in three major groups according to their respective fungicide resistance profile, i.e., (1) Wild type, sensitive to all seven fungicides reported previously; (2) Phenotype QoI^R^Bos^R^Ani^R^Ben^R^Dic^R^, resistant (R) to five fungicides; and (3) Phenotype Hyd^R^QoI^R^Bos^R^Ani^R^Phen^R^Ben^R^Dic^R^, resistant to all seven fungicides tested.

### Nucleic acid and library preparation—sequencing

High quality genomic DNA was extracted from 10 selected single-spore strains of each group (30 in total) by applying a CTAB based protocol (Chatzidimopoulos et al., 2014). Then, three different gene pools were generated containing the DNA of the selected strains according to the fungicide resistance profile. The quantity of DNA was estimated by picogreen (Invitrogen) method using Victor 3 fluorometry. The condition of DNA was checked by a gel electrophoresis method and the purity was assessed on a NanoDrop instrument.

Library preparation performed with the TruSeq Nano DNA Kit (Illumina) with target insert size of 550 bp and read lengths of 101 bp using 200 ng input gDNA. Whole genome sequenced on Illumina HiSeq 2000 with paired-end libraries generated for each of the three fungal genomes (Macrogen INC, 10F, 254 Beotkkot-ro, Geumcheon-gu, Seoul, Rep. of Korea). Fragmented DNA was cleaned up and the overhangs converted into blunt ends using End Repair Mix 2 (Illumina). In order to verify the size of PCR enriched fragments, the template size distribution was checked on a Agilent Technologies 2100 Bioanalyzer using a DNA 1000 chip. The DNA sequence of each cluster on a flow cell determined with the TruSeq SBS Kit v3 (Illumina). The generation of raw data performed with the HiSeq Control Software v2.2 (Illumina).

### Sequence clean-up, alignment, and variant calling

In order to consistently apply quality and adapter trimming to the sequences, the Cutadapt and FastQC tools were applied through the Trim Galore! wrapper application (Andrews, 2010; Martin, 2011; Krueger, 2012). The sequences were consequently aligned on the *B. cinerea* B05.10 reference genome, as retrieved from Fungi Ensembl (http://fungi.ensembl.org/Botrytis_cinerea/Info/Index). After building the reference index files the reads were aligned to the reference genome by using Bowtie 2 (Langmead and Salzberg, 2012) and the produced alignments were parsed using SAMTools (Li et al., 2009). In order to identify potential SNPs and INDELs further analysis was performed using GATK's UnifiedGenotyper (DePristo et al., 2011) and SnpEff v 4.2 (Cingolani et al., 2012).

## Results

A total of 12.8, 13, and 11.2 GB sequence data was obtained from the wild type (UTH.PPL.WT5), the 5-resistance variant (UTH.PPL.CR55), and the 7-resistance variant (UTH.PPL.MDR7) respectively. Low quality reads and adapter sequences were removed using FastQC and Cutadapt tools. After quality assessment, there was a loss of ~0.53, 0.45, and 0.46%, respectively (i.e., high quality sequences). Detailed information of the aligned reads is summarized in Table 1. The average coverage of the data across the reference genome was calculated using the samtools depth tool, and was found to be 95.9224, 108.681, and 87.2708 respectively. Moreover, the mapping rate of the three samples was approximately 80%, with the highest rate of 84.1% evident in UTH.PPL.CR55 and the lowest rate of 76.9 % in UTH.PPL.WT5. The datasets submitted to NCBI (raw reads) include the genome sequences of all three samples in FASTA format and can be accessed *via* the accession numbers reported in Table 1. Users can download and use the data freely for research purpose only with acknowledgment to us and quoting this paper as reference to the data.

**Table 1:** **Genome ID and overview of the three sample alignments against the ***Botrytis cinerea*** B05.10 reference genome**.

**Name**		**Attributes**		
Samples code		UTH.PPL.WT5	UTH.PPL.CR55	UTH.PPL.MDR7
NCBI bioproject ID		PRJNA307302		
NCBI biosample ID		SAMN04377718	SAMN04492128	SAMN04492129
NCBI SRA Accession No.		SRX1509486	SRX1592009	SRX1592010
Left reads	input	26,205,252	26,611,041	22,788,876
	mapped	20,887,799 (79.7%)	23,126,217 (86.9%)	18,628,763 (81.7%)
	have multiple alignments	361,717 (1.7%)	154,650 (0.7%)	89,293 (0.5%)
Right reads	input	26,205,252	26,611,041	22,788,876
	mapped	19,391,423 (74.0%)	21,649,707 (81.4%)	17,366,172 (76.2%)
	have multiple alignments	339,787 (1.8%)	147,450 (0.7%)	84,998 (0.5%)
Overall read mapping rate		76.9%	84.1%	79.0%
Aligned pairs		17,487,181	20,178,584	15,683,859
Multiple alignments		287,875 (1.6%)	136,479 (0.7%)	74,991 (0.5%)
Discordant alignments		165,381 (0.9%)	109,376 (0.5%)	57,563 (0.4%)
Concordant pair alignment rate		66.1%	75.4%	68.6%

## Author contributions

This project was planned and executed by MC under the supervision of PD. The data processing performed jointly by both FP and EM. IG, PM, and EV assisted substantially on the technical part of this work.

## Funding

The research project was funded under the Action “Research & Technology Development Innovation projects (AgroETAK),” MIS 453350, in the framework of the Operational Program “Human Resources Development.” It was co-funded by the European Social Fund and by National Resources through the National Strategic Reference Framework 2007–2013 (NSRF 2007–2013) coordinated by the Hellenic Agricultural Organization “DEMETER”/Institute of Plant Breeding and Genetic Resources.

### Conflict of interest statement

The authors declare that the research was conducted in the absence of any commercial or financial relationships that could be construed as a potential conflict of interest. The reviewer LP and handling Editor declared their shared affiliation, and the handling Editor states that the process nevertheless met the standards of a fair and objective review.
